# Point-of-care testing in Paediatric settings in the UK and Ireland: a cross-sectional study

**DOI:** 10.1186/s12873-021-00556-7

**Published:** 2022-01-11

**Authors:** Meenu Pandey, Mark D. Lyttle, Katrina Cathie, Alasdair Munro, Thomas Waterfield, Damian Roland, On behalf of GAPRUKI, PERUKI, Adrian Boyle, Peter Heinz, Shrouk Messahel, Dan Hawcutt, Caroline Ponmani, Chris Bird, Deepthi Jyothish, Catherine Williams, Ronan O’Sullivan, Elizabeth Jones, Mark Lyttle, Nwanneka Sargant, James Ross, Michael Barrett, Sinead Harty, Turlough Bolger, David Coghlan, Patrick Fitzpatrick, Conor Hensey, Tim Hussan, Kate Charlick, William Verling, Peter Christian, Matthew Clark, Bhavni Shah, John Criddle, Ronny Cheung, Roger Alcock, Patrick Aldridge, Russell Peek, Mark Anderson, Elizabeth Herrieven, Katherine Jerman, Arshid Murad, Charlotte Brown, Andy Marshall, Fleur Cantle, Gavin Wilson, Alice Downes, Damian Roland, Srini Bandi, Adebayo Da-Costa, Ray Barry, Natasha De Vere, Clare Dieppe, Jane Evans, Gayle Hann, Clare Tipper, Bengisu Bassay, Dermot Dalton, Lauren Fraser, Chris Gough, Sharryn Gardner, Mark Tighe, Darren Ranasinghe, Simon Birch, Sharon Hall, Gareth Patton, Steve Turner, Emily Walton, Julie-Ann Maney, Tom Bourke, Manish Thakker, Gisela Robinson, Lizzie Starkey, Andrew Appelboam, Shye Wei Wong, Steven Foster, Louisa Pollock, Jen Browning, Katherine Potier, Kirsty Challen, Elizabeth Gilby, Lisa Kehler, Sebastian Gray, Shammi Ramlakhan, Niall Mullen, Jane Bayreuther, Katrina Cathie, Heather Jarman, Neil Thompson, Ami Parikh, Siba Paul, Sarah Trippick, Alastair Sutcliffe, Joanne Mulligan, Sophie Keers, Jeff Morgan, Michelle Jacobs, Mike Linney, Sarah Wilson, Erum Jamall

**Affiliations:** 1 Paediatric Emergency Medicine Leicester Academic (PEMLA) Group, Children’s Emergency Department, Leicester Royal Infirmary, Leicester, UK; 2 Emergency Department, Bristol Royal Hospital for Children, Bristol, UK; 3 Faculty of Health and Applied Sciences, University of the West of England, Bristol, UK; 4 Department of Child Health, University Hospital Southampton NHS Foundation Trust, Southampton, UK; 5 National Institute of Health Research Southampton Clinical Research Facility and Biomedical Research Centre, University Hospital Southampton NHS Foundation Trust, Southampton, UK; 6 Wellcome-Wolfson Institute for Experimental Medicine, Queen’s University Belfast, Belfast, UK; 7 Emergency Department, Children’s Health Ireland, Temple Street, Dublin, Ireland; 8 SAPPHIRE Group, Health Sciences, Leicester University, Leicester, UK

**Keywords:** Technology, Molecular biology, Health services research, Data collection

## Abstract

**Background:**

Point-of-care testing (POCT) is diagnostic testing performed at or near to the site of the patient. Understanding the current capacity, and scope, of POCT in this setting is essential in order to respond to new research evidence which may lead to wide implementation.

**Methods:**

A cross-sectional online survey study of POCT use was conducted between 6th January and 2nd February 2020 on behalf of two United Kingdom (UK) and Ireland-based paediatric research networks (Paediatric Emergency Research UK and Ireland, and General and Adolescent Paediatric Research UK and Ireland).

**Results:**

In total 91/109 (83.5%) sites responded, with some respondents providing details for multiple units on their site based on network membership (139 units in total). The most commonly performed POCT were blood sugar (137/139; 98.6%), urinalysis (134/139; 96.4%) and blood gas analysis (132/139; 95%). The use of POCT for Influenza/Respiratory Syncytial Virus (RSV) (45/139; 32.4%, 41/139; 29.5%), C-Reactive Protein (CRP) (13/139; 9.4%), Procalcitonin (PCT) (2/139; 1.4%) and Group A Streptococcus (5/139; 3.6%) and was relatively low. Obstacles to the introduction of new POCT included resources and infrastructure to support test performance and quality assurance.

**Conclusion:**

This survey demonstrates significant consensus in POCT practice in the UK and Ireland but highlights specific inequity in newer biomarkers, some which do not have support from national guidance. A clear strategy to overcome the key obstacles of funding, evidence base, and standardising variation will be essential if there is a drive toward increasing implementation of POCT.

**Supplementary Information:**

The online version contains supplementary material available at 10.1186/s12873-021-00556-7.

## Background

There is a need for clinicians to make accurate and timely decisions regarding emergency management of their patients. Laboratory tests are often used, in conjunction with clinical findings, to determine the most appropriate care pathway. Delays in obtaining and reporting urgent samples can lead to department crowding, protracted discharge times, and failure to deliver optimal patient care in emergency and acute care settings [1–3]. Point-Of-Care Testing (POCT) has the potential to provide rapid and accurate results that reduce such delays [4–6]. Potential additional benefits include improved clinical management, treatment adherence, and patient satisfaction [5] which must be balanced against the clinical significance of time gained. For example, in the context of an over crowded department with long waiting times does a 15 min time to result improve outcomes for patients? Also there are concerns regarding reliability and cost of POCT compared with centralised laboratory testing, and appropriate governance of POCT. There is currently no clearly described consensus approach to procurement, implementation and governance of POCT in the UK and Ireland; similar lack of consensus is evident in medical literature internationally [7–11].

However emerging evidence and ongoing research continue to evaluate the potential impact of POCT. Should these tests prove to have good clinical utility, they are likely to be incorporated into national guidance, and clinical practice, more widely. Developing a framework for the clinical use, implementation, and governance of POCT is therefore essential within the healthcare system, especially those aiming to prioritise same day emergency care. It remains the case that many Emergency Departments serve both adult and paediatric populations, and POCT may serve the needs of both patient groups. This works well for test common to both age categories (such as glucose) but perhaps not so well for respiratory testing which is anecdotally more common place in paediatric practice. As no survey of POCT has previously been performed across Children’s Acute and Emergency Care, it is important to establish whether this is truly the case, across a widely inclusive range of POCTs.

The primary aim of this study was to describe POCT in current use in acute paediatric settings across the UK and Ireland. Secondary objectives were to examine implementation, maintenance, funding and governance and further opinions on the introduction of new POCT including obstacles and enablers.

## Methods

This online cross sectional survey was conducted between 6th January and 2nd February 2020, and is reported in accordance with the Checklist for Reporting Results of Internet E-Surveys (CHERRIES [12]).

Existing literature on use of POCT in acute paediatric settings [9–11] informed the initial survey content, which was subsequently refined iteratively by the study team. In the absence of international guidance, content was finalised by consensus of the study team following external review, and prior to launch the survey underwent external piloting. This rationlised the number of questions and determined the range of POCTs to be surveyed. The survey was distributed to member sites of the General Adolescent and Paediatric Research in the UK & Ireland (GAPRUKI) and Paediatric Emergency Research in the UK & Ireland (PERUKI [13]) networks, with one response for each relevant emergency or acute paediatric unit requested from each network site (not all sites were members of both networks). Responses could be provided on behalf of Emergency Departments, Paediatric Assessment Units, Paediatric ward settings, and Urgent Care Centres.

For the purposes of this survey, POCT was defined as an investigative or diagnostic test utilised by staff in a clinical environment, for which results are available in a short time (within 30 min) to aid clinical decision making in that setting (i.e. not at a later date/time). Additional detail in this definition stated that they should be performed and interpreted by clinical staff caring for the patient, not sent elsewhere for other personnel to analyse and interpret, and should require no other interpretation (i.e. the result is binary, sequential or categorical).

The full survey, available in Appendix 1, included questions on availability of a range of POCT across clinical settings. Adaptive questioning was used, and where applicable, respondents were asked questions on personnel performing and interpreting tests, and POCT governance. All respondents were asked to provide the view of their site on the potential benefits and challenges presented by the concept of expanding POCT use, and were asked to describe any obstacles or enablers from previous experience of implementing POCT.

Responses were collected in Research Electronic Data Capture tools (REDCap [14, 15]), and were held on a secure University of Bristol server. We followed a standard framework; open for 4 weeks with non-responders sent reminders with 2 weeks to go, 1 week to go, and 48 h to go. Responses were analysed anonymously by the study team using Microsoft Excel version 16.42). Data are presented using descriptive statistics, including number and proportion for categorical variables.

As this survey study contained no patient level data, and was distributed using professional collaborative professional networks, ethical approval was unnecessary according to the Health Research Authority framework decision tool [16] .

## Results

**Fig. 1:**
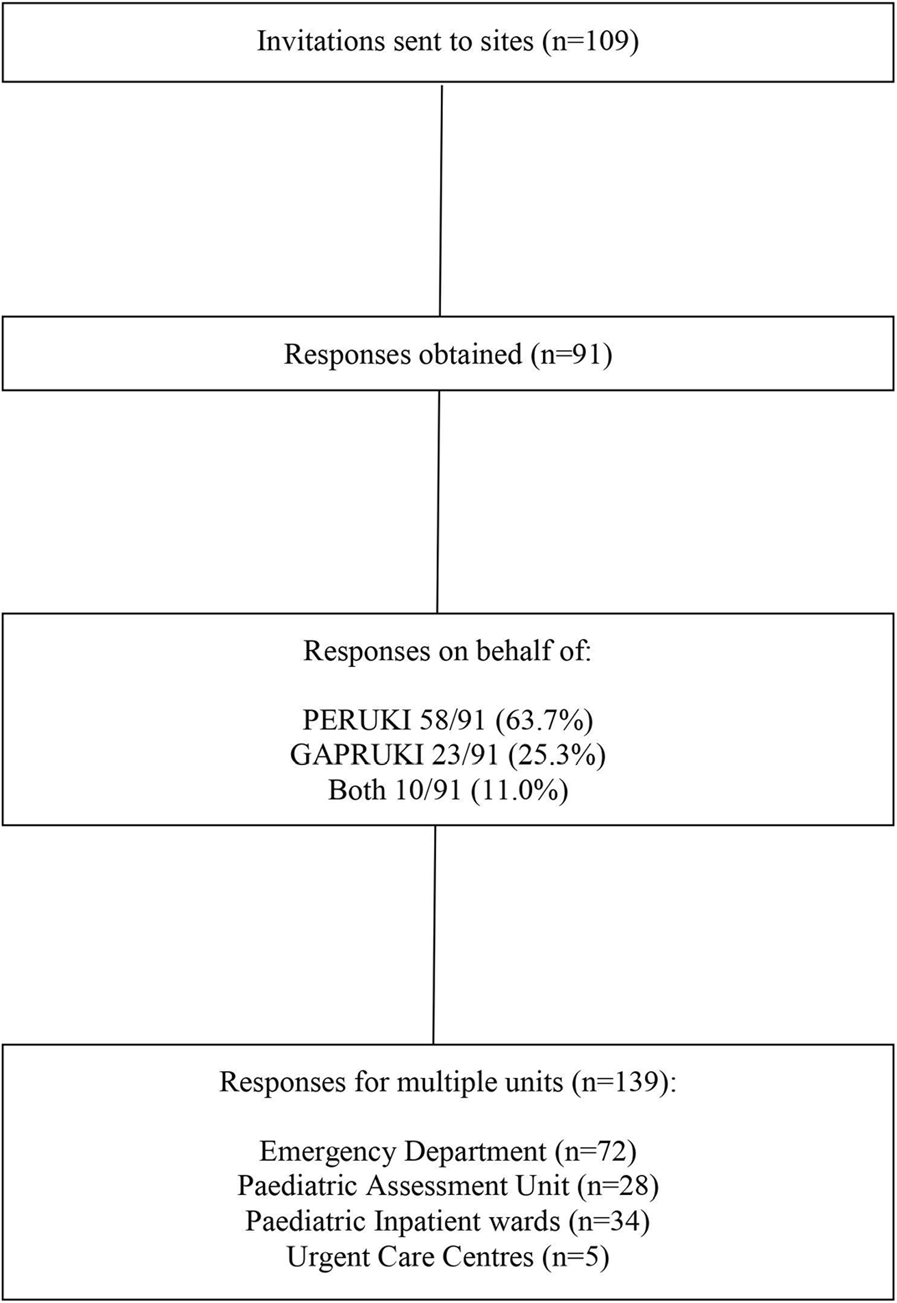
Flow diagram showing the flow of responses

In total, 109 invites were sent, to which there were 91 (83.5%) responses across 139 units (Fig. 1, each clinician could respond for more than one unit on their site), contributing data regarding 72 Emergency Departments (including Observation Units), 28 Paediatric Assessment Units, 34 Paediatric Inpatient wards, and five Urgent Care Centres.

There were a range of POCT available across sites, including those available in testing modalities analysing more than one marker simultaneously (for example, blood gas analysis variables) (Table 1). The most commonly performed POCT were blood sugar measurement (137/139; 98.6%), urinalysis (134/139; 96.4%) and blood gas analysis (132/139; 95%). In blood gas analysers, most sites had access to Lactate (127/139; 91.4%), pH/PaCO2/PaO2/Base Excess (126/139; 90.6%), Sodium/Potassium (126/139; 90.6%), Glucose (123/139; 88.5%) and Haemoglobin (116/139; 83.5%).

**Table 1:** Point Of Care Tests (POCT) done and investigations available on blood gas analysers

POCTs	Total (*n* = 139)	ED (*n* = 72)	PAU (*n* = 28)	UCC (*n* = 5)	Inpatient ward (*n* = 34)
Blood sugar	137/139 (98.6%)	72/72 (100%)	27/28 (96.4%)	5/5 (100%)	33/34 (97.1%)
Urinalysis	134/139 (96.4%)	70/72 (97.2%)	27/28 (96.4%)	5/5 (100%)	32/34 (94.1%)
Blood gas analysis	132/139 (95%)	72/72 (100%)	25/28 (89.3%)	3/5 (60%)	32/34 (94.1%)
Blood ketones	125/139 (89.9%)	66/72 (91.7%)	25/28 (89.3%)	3/5 (60%)	31/34 (91.2%)
Urinary Beta hCG	115/139 (82.7%)	66/72 (91.7%)	20/28 (71.4%)	5/5 (100%)	24/34 (70.6%)
Influenza (any)	45/139 (32.4%)	29/72 (40.3%)	7/28 (25%)	1/5 (20%)	8/34 (23.5%)
RSV	41/139 (29.5%)	24/72 (33.3%)	7/28 (25%)	1/5 (20%)	9/34 (26.5%)
Other	17/139 (12.2%)	15/72 (20.8%)	2/28 (7.1%)	0/5 (0%)	0/34 (0%)
CRP	13/139 (9.4%)	7/72 (9.7%)	3/28 (10.7%)	0/5 (0%)	3/34 (8.8%)
Group A Streptococcus	5/139 (3.6%)	3/72 (4.2%)	1/28 (3.6%)	0/5 (0%)	1/34 (2.9%)
Procalcitonin	2/139 (1.4%)	1/72 (1.4%)	0/28 (0%)	0/5 (0%)	1/34 (2.9%)
Blood gas analyser investigations available among units doing blood gases					
Lactate	127/139 (91.4%)	69/72 (95.8%)	25/28 (89.3%)	3/5 (60%)	30/34 (88.2%)
pH, PaCO2/PaO2, Base Excess	126/139 (90.6%)	69/72 (95.8%)	25/28 (89.3%)	3/5 (60%)	29/34 (85.3%)
Sodium/Potassium	126/139 (90.6%)	69/72 (95.8%)	24/28 (85.7%)	3/5 (60%)	30/34 (88.2%)
Glucose	123/139 (88.5%)	68/72 (94.4%)	23/28 (82.1%)	3/5 (60%)	29/34 (85.3%)
Haemoglobin	116/139 (83.5%	67/72 (93%)	21/28 (75%)	3/5 (60%)	25/34 (73.5%)
Calcium	107/139 (77%)	62/72 (86.1%)	19/28 (67.9%)	3/5 (60%)	23/34 (67.6%)
Bilirubin	46/139 (33.1%)	25/72 (34.7%)	11/28 (39.3%)	1/5 (20%)	9/34 (26.5%)
Phosphate	27/139 (19.2%)	18/72 (25%)	2/28 (7.1%)	1/5 (20%)	6/34 (17.6%)
Other:	23/139 (16.5%)	18/72 (25%)	3/28 (10.7%)	0/5 (0%)	2/34 (5.9%)

*ED* Emergency Department, *PAU* Paediatric Assessment Unit, *UCC* Urgent Care Centre, *hCG* human Chorionic Gonadotrophin, *RSV* Respiratory Syncytial Virus, *CRP* C-reactive protein

The use of Influenza/Respiratory Syncytial Virus (RSV) POCT were available in approximately one-third of sites (45/139; 32.4%, and 41/139; 29.5%, respectively), whilst availability of POCT for other biomarkers including C - reactive protein (CRP) (13/139; 9.4%), Group A Streptococcus (5/139; 3.6%) and Procalcitonin (PCT) (2/139; 1.4%) was markedly lower.

A description of staff types performing and interpreting tests is provided in Table 2; this is further quantified by clinical area in Supplementary Tables 5 and 6. As multiple staff groups could be selected for each POCT for each unit, there was a total of 2132 responses relating to staff roles in performance of POCT, and 3097 relating to actioning of results. Clinical nurses were the staff group most commonly responsible for POCT performance (705/2132; 33.1%) followed by Emergency Nurse Practitioners (ENP)/Advanced Nurse Practitioners (ANP) (435/2132, 20.4%) and junior doctors (385/2132; 18.1%). POCT were mostly acted on by senior non-consultants (736/3097; 23.8%), consultants (736/3097; 23.8%) and junior trainees (702/3097; 22.7%).

**Table 2:** Staff members responsible for performing and acting on POCT results

Staff members who perform each POCT								
	Clinical Nurse	Healthcare assistant	ENP/ACP	Junior Doctor		Consultant	Other	Total
Blood sugar	135/325 (41.5%)	2/325 (0.6%)	71/325 (21.8%)	61/325 (18.8%)		54/325 (16.6%)	2/325 (0.6%)	325 (100%)
Urinalysis	134/444 (30.2%)	87/444 (19.6%)	85/444 (19.1%)	74/444 (16.7%)		59/444 (13.3%)	5/444 (1.1%)	444 (100%)
Blood gas analysis	107/471 (22.7%)	46/471 (9.8%)	86/471 (18.3%)	121/471 (25.7%)		109/471 (23.1%)	2/471 (0.4%)	471 (100%)
Blood Ketones	120/292 (41.1%)	0/292 (0%)	70/292 (24%)	52/292 (17.8%)		47/292 (16.1%)	3/292 (1%)	292 (100%)
Urinary Beta HCG	115/350 (32.9%)	86/350 (24.6%)	70/350 (20%)	43/350 (12.3%)		34/350 (9.7%)	2/350 (0.6%)	350 (100%)
Influenza	45/105 (42.9%)	19/105 (18.1%)	22/105 (21%)	10/105 (9.5%)		9/105 (8.6%)	0/105 (0%)	105 (100%)
RSV	39/84 (46.4%)	14/84 (16.7%)	17/84 (20.2%)	7/84 (8.3%)		5/84 (6%)	2/84 (2.4%)	84 (100%)
CRP	6/43 (14%)	2/43 (4.7%)	11/43 (25.6%)	13/43 (30.2%)		11/43 (25.6%)	0/43 (0%)	43 (100%)
GAS	4/14 (28.6%)	3/14 (21.4%)	3/14 (21.4%)	2/14 (14.3%)		2/14 (14.3%)	0/14 (0%)	14 (100%)
Pct	0/4 (0%)	0/4 (0%)	0/4 (0%)	2/4 (50%)		2/4 (50%)	0/4 (0%)	4 (100%)
Total	705/2132 (33.1%)	259/2132 (12.1%)	435/2132 (20.4%)	385/2132 (18.1%)		332/2132 (15.6%)	16/2132 (0.8%)	2132 (100%)
Staff who are responsible for acting on POCT results								
	Clinical Nurse	Healthcare assistant	ENP/ACP	Junior Trainee (eg ST1–3)	Senior non-Consultant (eg ST4+)	Consultant	Other	Total
Blood sugar	97/601 (16.1%)	10/601 (1.7%)	93/601 (15.5%)	132/601 (22%)	135/601 (22.5%)	132/601 (22%)	2/601 (0.3%)	601 (100%)
Urinalysis	59/555 (10.6%)	13/555 (2.3%)	91/555 (16.4%)	128/555 (23.1%)	132/555 (23.8%)	130/555 (23.4%)	2/555 (0.4%)	555 (100%)
Blood gas analysis	33/493 (67.5%)	4/493 (0.8%)	80/493 (16.2%)	120/493 (24.3%)	127/493 (25.8%)	127/493 (25.8%)	2/493 (0.4%)	493 (100%)
Blood Ketones	62/522 (11.9%)	8/522 (1.5%)	86/522 (16.5%)	118/522 (22.6%)	124/522 (23.8%)	123/522 (23.6%)	1/522 (0.2%)	522 (100%)
Urinary Beta hCG	44/465 (9.5%)	8/465 (1.7%)	78/465 (16.8%)	109/465 (23.4%)	113/465 (24.3%)	111/465 (23.9%)	2/465 (0.4%)	465 (100%)
Influenza	34/206 (16.5%)	7/206 (3.4%)	35/206 (17%)	40/206 (19.4%)	45/206 (21.8%)	44/206 (21.4%)	1/206 (0.5%)	206 (100%)
RSV	32/181 (17.7%)	3/181 (1.7%)	29/181 (16%)	36/181 (19.9%)	40/181 (22.1%)	40/181 (22.1%)	1/181 (0.6%)	181 (100%)
CRP	0/48 (0%)	0/48 (0%)	10/48 (20.8%)	12/48 (25%)	13/48 (27.1%)	13/48 (27.1%)	0/48 (0%)	48 (100%)
GAS	0/19 (0%)	0/19 (0%)	4/19 (21.1%)	5/19 (26.3%)	5/19 (26.3%)	5/19 (26.3%)	0/19 (0%)	19 (100%)
Pct	0/7 (0%)	0/7 (0%)	1/7 (14.3%)	2/7 (28.6%)	2/7 (28.6%)	2/7 (28.6%)	0/7 (0%)	7 (100%)
Total	361/3097 (11.7%)	53/3097 (1.7%)	507/3097 (16.4%)	702/3097 (22.7%)	736/3097 (23.8%)	727/3097 (23.5%)	11/3097 (0.4%)	3097 (100%)

*POCT* Point-of-Care Test, *ENP* Emergency nurse practitioner, *ANP* Advanced nurse practitioner, *ST* Specialty trainee, *hCG* human Chorionic Gonadotrophin, *RSV* Respiratory Syncytial Virus, *CRP* C-reactive protein, *GAS* Group A Streptococcus, *Pct* Procalcitonin

Information regarding the non-clinical utilisation of POCT is provided in Table 3, with 561 responses related to governance, and 677 responses related to data storage.

**Table 3:** Responsibility for POCT governance and documentation/storage

	Who is responsible for POCT governance?					How and where are POCT results stored?						
	Laboratory team take full responsibility for governance	Clinical staff take some responsibility for governance, in conjunction with laboratory teams	Other	Not applicable	Total	Handwritten in clinical record	Manual entry in electronic record	Printed out and stuck in record	Auto upload to electronic system	Other	Not applicable	Total
Blood sugar	25/99 (25.3%)	69/99 (69.7%)	5/99 (5.1%)	0/99 (0%)	99 (100%)	54/111 (48.6%)	32/111 (28.8%)	12/111 (10.8%)	13/111 (11.7%)	0/111 (0%)	0/111 (0%)	111 (100%)
Urinalysis	22/94 (23.4%)	66/94 (70.2%)	5/94 (5.3%)	1/94 (1.1%)	94 (100%)	37/111 (33.3%)	27/111 (24.3%)	42/111 (37.8%)	5/111 (4.5%)	0/111 (0%)	0/111 (0%)	111 (100%)
Blood gas	53/99 (53.5%)	43/99 (43.4%)	3/99 (3%)	0/99 (0%)	99 (100%)	23/142 (16.2%)	12/142 (8.5%)	64/142 (45.1%)	42/142 (29.6%)	1/142 (0.7%)	0/142 (0%)	142 (100%)
Blood Ketones	23/92 (25%)	63/92 (68.5%)	6/92 (6.5%)	0/92 (0%)	92 (100%)	52/104 (52%)	32/104 (30.8%)	9/104 (8.7%)	11/104 (10.6%)	0/104 (0%)	0/104 (0%)	104 (100%)
Urinary Beta hCG	17/82 (20.7%)	58/82 (70.7%)	4/82 (4.9%)	3/82 (3.7%)	82 (100%)	36/91 (39.6%)	25/91 (27.5%)	27/91 (29.7%)	3/91 (3.3%)	0/91 (0%)	0/91 (0%)	91 (100%)
Influenza	20/41 (48.8%)	21/41 (51.2%)	0/41 (0%)	0/41 (0%)	41 (100%)	17/50 (34%)	8/50 (16%)	11/50 (22%)	13/50 (26%)	1/50 (2%)	0/50 (0%)	50 (100%)
RSV	16/34 (47.1%)	18/34 (52.9%)	0/34 (0%)	0/34 (0%)	34 (100%)	14/45 (31.1%)	8/45 (17.8%)	8/45 (17.8%)	14/45 (31.1%)	1/45 (2.2%)	0/45 (0%)	45 (100%)
CRP	8/12 (66.7%)	4/12 (33.3%)	0/12 (0%)	0/12 (0%)	12 (100%)	4/15 (26.7%)	1/15 (6.7%)	5/15 (33.3%)	5/15 (33.3%)	0/15 (0%)	0/15 (0%)	15 (100%)
Group A Streptococcus	1/5 (20%)	2/5 (40%)	2/5 (40%)	0/5 (0%)	5 (100%)	3/4 (75%)	1/4 (25%)	0/4 (0%)	0/4 (0%)	0/4 (0%)	0/4 (0%)	4 (100%)
Procalcitonin	1/3 (33.3%)	2/3 (66.7%)	0/3 (0%)	0/3 (0%)	3 (100%)	1/4 (25%)	0/4 (0%)	2/4 (50%)	1/4 (25%)	0/4 (0%)	0/4 (0%)	4 (100%)
Total; n (n% of total for each section)	186/561 (33.2%)	346/561 (61.7%)	25/561 (4.5%)	4/561 (0.7%)	561 (100%)	241/677 (35.6%)	146/677 (21.6%)	180/677 (26.6%)	107/677 (15.8%)	3/677 (0.4%)	0/677 (0%)	677 (100%)

*POCT* Point-of-Care Test, *hCG* human Chorionic Gonadotrophin, *RSV* Respiratory Syncytial Virus, *CRP* C-reactive protein

Most commonly, clinical staff took some responsibility for POCT governance in conjunction with laboratory teams (346/561; 61.7%), followed by laboratory teams taking full responsibility (186/561; 33.2%). The POCT most likely to come under shared responsibility were urinary human Chorionic Gonadotropin (hCG) (58/82; 70.7%), urinalysis (66/94; 70.2%), blood sugar (69/99; 69.7%) and blood ketones (63/92; 68.5%). POCT which give multiple results (such as blood gases) were primarily managed by laboratory teams (53/99; 53.5%,).

The most common method of data storage was handwritten notes in clinical records (241/677; 35.6%) followed by printouts attached to medical records (180/677; 26.6%) and manual entry (146/677; 21.6%). Automatic uploading to electronic systems occurred in only 15.8% of responses (107/677).

Obstacles to POCT introduction (158 responses), and nature of funding sources (98 responses), are displayed in Table 4. The most commonly reported obstacles were difficulties with funding (72/158; 45.6%), lack of evidence (33/158; 20.9%) and issues with POCT governance (20/158; 12.7%). POCT were typically funded as part of an ongoing service with sustainable long-term funding (81/98; 82.7%). Other methods of funding included temporary funds as part of a service evaluation (10/98; 10.2%), or charitable funding and/or donations (5/98; 5.1%).

**Table 4:** Obstacles to introduction of POCT and sources of current POCT funding in units

**Obstacles currently existing to the use of POCT**	**n**	**% of 158 responses**
Difficulties with funding	72	45.6%
Evidence is lacking for POCT	33	20.9%
Nobody will take responsibility for the governance of the test	20	12.7%
Nobody has time to perform the quality control testing	16	10.1%
Other	12	7.6%
Nobody has time to run the test	5	3.2%
**Total**	**158**	**100%**
**Sources of current POCT funding**		**% of 98 responses**
All funded as part of ongoing service with sustainable longterm funding	81	82.7%
Some funded using temporary fund as part of a service evaluation	10	10.2%
Some funded through charitable funding and/or donations	5	5.1%
Some funded as part of an industry sponsored trial	1	1%
Other	1	1%
**Total**	**98**	**100%**

## Discussion

We have demonstrated a diverse range of POCT in use in Children’s Emergency Departments and Assessment units. Some POCT, such as blood sugar testing, blood ketone testing, blood gas analysis and urinalysis are fairly commonplace, whilst “newer” POCT such as CRP and Procalcitonin were uncommon. Despite this penetrance of POCT into acute units, we have also identified wide variation in their governance, and usage processes. The challenge of identifying the optimal governance of POCT appears to hinder their implementation, as do a current lack of evidence, and cost; these elements are particularly important when considering introducing POCT to national guidance if emerging evidence supports their use.

Respiratory POCT were utilised in under half the responding units, with Respiratory Syncytial Virus (RSV) and influenza usage at 33.3 and 40.3% respectively. POCT for RSV has previously been evaluated and found to be a safe, cost-effective, and efficient way to improve bed management [17]. The lack of widespread utilisation is, therefore, perhaps surprising. However, some centres cohort infants with bronchiolitis based on symptoms, in which case POCT may conversely delay admission. A formal evaluation of these two approaches would help determine the utility of POCT in this situation. However, it must be recognised that determining viral aetiology is useful for public health surveillance and it is likely that ward based testing will still need to occur.

For influenza, one study of the use of POCT in febrile children showed no difference in physician management, cost, or length of stay in the paediatric Emergency Department (ED) [7]. However, a positive POCT for influenza was associated with a significant reduction in urine and blood cultures being sent for febrile children [18].

Some studies have recommended use of rapid streptococcal A infection testing for patients with a sore throat, citing reduction of antimicrobial use [19, 20]. However, in England, this is not routinely recommended by National Institute of Health and Care Excellence (NICE) guidance, as this approach is unlikely to be cost-effective. Their limited role in improving antimicrobial prescribing and stewardship, as well as patient outcomes, when compared to clinical scores alone [21] is the likely reason for the low use in surveyed sites.

In relation to biomarkers for infection, a systematic review and cost-effectiveness analysis evaluated whether Procalcitonin testing was helpful in guiding antibiotic therapy for sepsis in intensive care and ED settings [22, 23]. This concluded that addition of a Procalcitonin based algorithm to antibiotic guidance could be useful in reducing antibiotic exposure and length of hospital stay safely in adults [22]. Clinicians might infer that similar results may be seen in children, however high quality evidence is lacking, and further research is needed on the utility of Procalcitonin in this domain.

The most common source of funding for POCT was sustainable long term funding as part of an ongoing service commitment for well-established POCT; these included blood gas analysis, urinalysis and blood sugar testing, and are routinely used in > 90% of departments. The newer POCT were more likely to be funded using temporary funds as part of a service evaluation, through charitable funding and/ or donations or as part of an industry sponsored trial.

Given our findings regarding the challenges of implementing new POCT, robust evidence for patient benefit is required in order to provide a clear case to justify National Health Service (NHS) spending on the testing equipment and consumables required to use in routine clinical practice.

We acknowledge the limitations of a survey based qualitative study but believe our response rate and reach were sufficient to avoid significant bias. We acknowledge that some operator training and quality control have been identified as barriers to adoption and we didn’t ask focused questions on these. This was a point-in-time survey and it is likely that the situation in many departments may be different now than at the time the survey was undertaken. However we hope our identification of domains of interests will be useful for future evaluation and research.

In summary, if new POCT are to be introduced, it is vital that all stakeholders are involved in the decision making including clinical and laboratory teams, patients, regulatory authorities and insurers. Sustainability will depend on sound evidence base and financial viability [24].

## Conclusions

The use of POCT for blood glucose, blood gas, urinalysis and blood ketones is widespread among UK Children’s Emergency Departments and Assessment units, however newer biomarkers tests are used less often, including those for pathogen identification. Variation exists both in unit practices, and the governance of POCT. A clear strategy to overcome the key obstacles of funding, evidence base, and standardising variation will be essential if there is a drive toward increasing implementation of POCT.

## Supplementary Information

**Additional file 1.**

**Additional file 2.**

## Data Availability

All data generated or analysed during this study are included in this published article [and its supplementary information files].

## Abbreviations

GAPRUKI: General and Adolescent Paediatric Research UK and Ireland
PERUKI: Paediatric Emergency Research UK and Ireland
PEMLA group: Paediatric Emergency Medicine Leicester Academic Group
SAPPHIRE Group: Social Science Applied to Healthcare Improvement Research
POCT: Point-Of-Care Testing
UK: United Kingdom
RSV: Respiratory Syncytial Virus
CRP: C - Reactive Protein
PCT: Procalcitonin
CHERRIES: Checklist for Reporting Results of Internet E-Surveys
REDCap: Research Electronic Data Capture tools
ENP: Emergency Nurse Practitioners
ANP: Advanced Nurse Practitioners
hCG: human Chorionic Gonadotropin
ED: Emergency Department
NICE: National Institute of Health and Care Excellence
NHS: National Health Service
PAU: Paediatric Assessment Unit
UCC: Urgent Care Centre
ST: Specialty trainee
GAS: Group A Streptococcus
IP: Inpatient
