# Supplementary material for: Point-of-care testing in Paediatric settings in the UK and Ireland: a cross-sectional study
Source: BMC Emerg Med. 2022 Jan 11;22:6. doi: 10.1186/s12873-021-00556-7 (PMC8753865; doi:10.1186/s12873-021-00556-7)
Supplement: Supplementary file 1 — Additional file 1. [file 12873_2021_556_MOESM1_ESM.pdf]

Point of care testing survey

Increasingly EDs and acute assessment units use point of care (POC) testing to aid clinical decision making. Blood gas analysers and rapid viral antigen testing (eg for RSV) are common, but POC biomarker tests (eg CRP/PCT) are less widespread. This short survey aims to find out which POC tools are used to evaluate children in urgent, acute and emergency care in the UK and Ireland.

Survey instructions:

For each POC test please mark where in your hospital it is available for clinical decision making. If the POC machine is not in the clinical area but the test can be run and the result brought back, please mark where the test result is utilised - not where the POC machine is based.

Definition of POC testing for this survey:

An investigative/diagnostic test utilised by staff in a clinical environment, for which results are available in a short time (< 30 minutes) to aid clinical decision making in that setting (ie not at a later date/time)

Should be performed and interpreted by clinical staff caring for the patient, not sent elsewhere for other personnel to analyse and interpret

Requires no other interpretation (ie the result is binary, sequential or categorical) - and so investigations such as ECG and ultrasound are not included, as this requires the user to make a subjective interpretation of the result

Data management:

All responses are held on a secure University of Bristol server, and analysed anonymously by the study team. We ask for your email and site in case we need to clarify any answers, and to make sure we can recognise you on any outputs - this also means we will stop nagging you when you've completed it. Your consent to participate will be implied by completing this survey, - you will not be added to any mailing lists as a result of completing this, nor will your information be shared with any external parties.

Please tell us your name

____

Please share your email

____
(Including this should hopefully trigger your certificate being sent - please let us know if it doesn't arrive....)

Please select your site

◯ Addenbrooke's Hospital
◯ Alder Hey Children's Hospital NHS Foundation Trust
◯ Barking, Havering & Redbridge University Hospitals NHS Trust
◯ Birmingham Children's Hospital
◯ Blackrock Clinic, Dublin
◯ Bon Secours Hospital, Cork
◯ Bristol Royal Hospital for Children
◯ Chelsea and Westminster Hospital
◯ Children's Health Ireland at Crumlin
◯ Children's Health Ireland at Tallaght
◯ Children's Health Ireland at Temple Street
◯ City Hospitals Sunderland NHS Foundation Trust
◯ Cork University Hospital
◯ Countess of Chester NHS Foundation Trust
◯ County Durham & Darlington NHS Foundation Trust
◯ Craigavon Area Hospital
◯ Derriford Hospital
◯ Dorset County Hospital
◯ East Kent Hospital
◯ East Sussex NHS Health Trust
◯ Evelina London Children's Hospital
◯ Gloucestershire Hospitals NHS Foundation Trust
◯ Great Ormond Street Hospital, London
◯ Great North Children's Hospital, Newcastle Upon Tyne
◯ Grimsby Hospital
◯ Hampshire Hospitals Foundation Trust
◯ Harrogate & District NHS Foundation Trust
◯ Hoddesden Hospital
◯ Hull Royal Infirmary
◯ Ipswich Hospital
◯ James Cook University Hospital
◯ John Radcliffe Hospital, Oxford
◯ King's College Hospital, London
◯ Kingston Hospital
◯ Leeds General Infirmary
◯ Leicester Royal Infirmary
◯ Limerick Hospital, Ireland
◯ Lister Hospital, Stevenage
◯ Maidstone and Tunbridge Wells NHS Trust
◯ Medway Maritime Hospital
◯ Mercy University Hospital, Cork, Ireland
◯ Morriston Hospital
◯ Musgrove Park Hospital
◯ North Manchester General Hospital
◯ North Middlesex Hospital
◯ Northampton General Hospital
◯ Northern Devon Healthcare NHS Trust
◯ Northwick Park Hospital
◯ Nottingham University Hospitals NHS Trust
◯ Ormskirk & District General Hospital
◯ Poole Hospital
◯ Prince Charles Hospital
◯ Queen Alexandra Hospital
◯ Queen Elizabeth Hospital, Woolwich
◯ Raigmore Hospital, Inverness
◯ Royal Aberdeen Children's Hospital
◯ Royal Alexandra Children's Hospital
◯ Royal Belfast Hospital for Sick Children
◯ Royal Berkshire NHS Foundation Trust
◯ Royal Bournemouth Hospital
◯ Royal Derby Hospital
◯ Royal Devon and Exeter Hospital
◯ Royal Free Hospital
◯ Royal Glamorgan Hospital
◯ Royal Hospital for Children, Glasgow
◯ Royal Hospital for Sick Children, Edinburgh
◯ Royal Manchester Children's Hospital

◯ Royal Preston Hospital
◯ Royal United Hospital, Bath
◯ Royal Wolverhampton NHS Trust
◯ Salisbury NHS Foundation Trust
◯ Sheffield Children's Hospital
◯ Shrewsbury and Telford
◯ Southampton Children's Hospital
◯ St George's Hospital
◯ St Mary's Hospital
◯ The Royal London Hospital
◯ Torbay and South Devon NHS Foundation Trust
◯ Trinity College, Dublin
◯ University College Hospital London
◯ University Hospital Crosshouse
◯ University Hospital Lewisham
◯ University Hospital of Wales, Cardiff
◯ Wakefield Hospital
◯ Watford General Hospital
◯ Waterford Regional Hospital, Ireland
◯ Western Sussex Hospitals NHS Trust
◯ Wexham Park Hospital
◯ Whittington Health NHS Trust
◯ Other
(Tip: search for your site using free text rather than dropdown...)

If you answered other, please tell us the name of your site

____

Which network are you answering on behalf of?

☐ PERUKI
☐ GAPRUKI
☐ Both
☐ Neither

Which department(s) will you provide answers for on point of care testing (POCT)?

☐ Emergency Department (including ED observation unit)
☐ Paediatric Assessment Unit
☐ Urgent Care Centre
☐ Paediatric inpatient ward(s)

Emergency Department (including ED observation unit)

Which POCTs do you perform in the ED?

☐ CRP
☐ Procalcitonin
☐ RSV
☐ Influenza (any)
☐ Group A Strep
☐ Blood gas analysis
☐ Urinalysis
☐ Urinary Beta HCG
☐ Blood sugar
☐ Blood ketones
☐ Other

Please select which of the following are available on your ED blood gas analyser

- ☐ pH, PaCO2/PaO2, Base Excess
- ☐ Haemoglobin
- ☐ Glucose
- ☐ Lactate
- ☐ Bilirubin
- ☐ Sodium/Potassium
- ☐ Calcium
- ☐ Phosphate
- ☐ Other

Please tell us which other variables are available on this blood gas analyser

____

Please tell us about any other POCT available in the ED

____

Please select here all staff members who perform each POCT in the ED

	Clinical Nurse	Healthcare assistant	ENP/ACP	Junior Doctor	Consultant	Other
CRP	☐	☐	☐	☐	☐	☐
Procalcitonin	☐	☐	☐	☐	☐	☐
RSV	☐	☐	☐	☐	☐	☐
Influenza	☐	☐	☐	☐	☐	☐
Group A Strep	☐	☐	☐	☐	☐	☐
Blood gas	☐	☐	☐	☐	☐	☐
Urinalysis	☐	☐	☐	☐	☐	☐
Urinary BHCG	☐	☐	☐	☐	☐	☐
Blood sugar	☐	☐	☐	☐	☐	☐
Blood ketones	☐	☐	☐	☐	☐	☐

For those where you answered other, please tell us who does these

____

Please select here all staff who are responsible for acting on POCT results in the ED

	Clinical Nurse	Healthcare assistant	ENP/ACP	Junior Trainee (eg ST1-3)	Senior non-Consultant (eg ST4+)	Consultant	Other
CRP	☐	☐	☐	☐	☐	☐	☐
Procalcitonin	☐	☐	☐	☐	☐	☐	☐
RSV	☐	☐	☐	☐	☐	☐	☐
Influenza	☐	☐	☐	☐	☐	☐	☐
Group A Strep	☐	☐	☐	☐	☐	☐	☐
Blood gas	☐	☐	☐	☐	☐	☐	☐
Urinalysis	☐	☐	☐	☐	☐	☐	☐
Urinary BHCG	☐	☐	☐	☐	☐	☐	☐
Blood sugar	☐	☐	☐	☐	☐	☐	☐
Blood ketones	☐	☐	☐	☐	☐	☐	☐

For those where you answered other, please tell us who has responsibility for acting on these

____

Paediatric Assessment Unit (PAU)

Which POCTs do you perform in the PAU?

☐ CRP
☐ Procalcitonin
☐ RSV
☐ Influenza (any)
☐ Group A Strep
☐ Blood gas analysis
☐ Urinalysis
☐ Urinary Beta HCG
☐ Blood sugar
☐ Blood ketones
☐ Other

Please select which of the following are available on your PAU blood gas analyser

☐ pH, PaCO2/PaO2, Base Excess
☐ Haemoglobin
☐ Glucose
☐ Lactate
☐ Bilirubin
☐ Sodium/Potassium
☐ Calcium
☐ Phosphate
☐ Other

Please tell us which other variables are available on this blood gas analyser

____

Please tell us about any other POCT available in the PAU

____

Please select here all staff members who perform each POCT in the PAU

	Clinical Nurse	Healthcare assistant	ENP/ACP	Junior Doctor	Consultant	Other
CRP	☐	☐	☐	☐	☐	☐
Procalcitonin	☐	☐	☐	☐	☐	☐
RSV	☐	☐	☐	☐	☐	☐
Influenza	☐	☐	☐	☐	☐	☐
Group A Strep	☐	☐	☐	☐	☐	☐
Blood Gas	☐	☐	☐	☐	☐	☐
Urinalysis	☐	☐	☐	☐	☐	☐
Urinary BHCG	☐	☐	☐	☐	☐	☐
Blood sugar	☐	☐	☐	☐	☐	☐
Blood ketones	☐	☐	☐	☐	☐	☐

Where you've selected other, please tell us who does these

____

Please select here all staff who are responsible for acting on POCT results in the PAU

	Clinical Nurse	Healthcare assistant	ENP/ACP	Junior Trainee (eg ST1-3)	Senior non-Consultant (eg ST4+)	Consultant	Other
CRP	☐	☐	☐	☐	☐	☐	☐
Procalcitonin	☐	☐	☐	☐	☐	☐	☐
RSV	☐	☐	☐	☐	☐	☐	☐
Influenza	☐	☐	☐	☐	☐	☐	☐
Group A Strep	☐	☐	☐	☐	☐	☐	☐
Blood gas	☐	☐	☐	☐	☐	☐	☐
Urinalysis	☐	☐	☐	☐	☐	☐	☐
Urinary BHCG	☐	☐	☐	☐	☐	☐	☐
Blood sugar	☐	☐	☐	☐	☐	☐	☐
Blood ketones	☐	☐	☐	☐	☐	☐	☐

For those where you answered other, please tell us who has responsibility for acting on these

____

Urgent Care Centre (UCC)

Which POCTs do you perform in the UCC?

- ☐ CRP
- ☐ Procalcitonin
- ☐ RSV
- ☐ Influenza (any)
- ☐ Group A Strep
- ☐ Blood gas analysis
- ☐ Urinalysis
- ☐ Urinary Beta HCG
- ☐ Blood sugar
- ☐ Blood ketones
- ☐ Other

Please select which of the following are available on your UCC blood gas analyser

- ☐ pH, PaCO2/PaO2, Base Excess
- ☐ Haemoglobin
- ☐ Glucose
- ☐ Lactate
- ☐ Bilirubin
- ☐ Sodium/Potassium
- ☐ Calcium
- ☐ Phosphate
- ☐ Other

Please tell us which other variables are available on this blood gas analyser

____

Please tell us about any other POCT available in the UCC

____

Please select here all staff members who perform each POCT in the UCC

	Clinical Nurse	Healthcare assistant	ENP/ACP	Junior Doctor	Consultant	Other
CRP	☐	☐	☐	☐	☐	☐
Procalcitonin	☐	☐	☐	☐	☐	☐
RSV	☐	☐	☐	☐	☐	☐
Influenza	☐	☐	☐	☐	☐	☐
Group A Strep	☐	☐	☐	☐	☐	☐
Blood gas	☐	☐	☐	☐	☐	☐
Urinalysis	☐	☐	☐	☐	☐	☐
Urinary BHCG	☐	☐	☐	☐	☐	☐
Blood sugar	☐	☐	☐	☐	☐	☐
Blood ketones	☐	☐	☐	☐	☐	☐

Where you've answered other, please tell us who does these

____

Please select here all staff who are responsible for acting on POCT results in the UCC

	Clinical Nurse	Healthcare assistant	ENP/ACP	Junior Trainee (eg ST1-3)	Senior non-Consultant (eg ST4+)	Consultant	Other
CRP	☐	☐	☐	☐	☐	☐	☐
Procalcitonin	☐	☐	☐	☐	☐	☐	☐
RSV	☐	☐	☐	☐	☐	☐	☐
Influenza	☐	☐	☐	☐	☐	☐	☐
Group A Strep	☐	☐	☐	☐	☐	☐	☐
Blood gas	☐	☐	☐	☐	☐	☐	☐
Urinalysis	☐	☐	☐	☐	☐	☐	☐
Urinary BHCG	☐	☐	☐	☐	☐	☐	☐
Blood sugar	☐	☐	☐	☐	☐	☐	☐
Blood ketones	☐	☐	☐	☐	☐	☐	☐

For those where you answered other, please tell us who has responsibility for acting on these

____

Inpatient wards

Which POCTs do you perform in your inpatient ward(s)?

- ☐ CRP
- ☐ Procalcitonin
- ☐ RSV
- ☐ Influenza (any)
- ☐ Group A Strep
- ☐ Blood gas analysis
- ☐ Urinalysis
- ☐ Urinary Beta HCG
- ☐ Blood sugar
- ☐ Blood ketones
- ☐ Other

Please select which of the following are available on your inpatient ward blood gas analyser

- ☐ pH, PaCO2/PaO2, Base Excess
- ☐ Haemoglobin
- ☐ Glucose
- ☐ Lactate
- ☐ Bilirubin
- ☐ Sodium/Potassium
- ☐ Calcium
- ☐ Phosphate
- ☐ Other

Please tell us which other variables are available on this blood gas analyser

____

Please tell us about any other POCT available in the inpatient wards

____

Please select here all staff members who perform each POCT in the Inpatient ward

	Clinical Nurse	Healthcare assistant	ENP/ACP	Junior Doctor	Consultant	Other
CRP	☐	☐	☐	☐	☐	☐
Procalcitonin	☐	☐	☐	☐	☐	☐
RSV	☐	☐	☐	☐	☐	☐
Influenza	☐	☐	☐	☐	☐	☐
Group A Strep	☐	☐	☐	☐	☐	☐
Blood gas	☐	☐	☐	☐	☐	☐
Urinalysis	☐	☐	☐	☐	☐	☐
Urinary BHCG	☐	☐	☐	☐	☐	☐
Blood sugar	☐	☐	☐	☐	☐	☐
Blood ketones	☐	☐	☐	☐	☐	☐

Where you've answered other, please tell us who does these

____

Please select here all staff who are responsible for acting on POCT results in the Inpatient Wards

	Clinical Nurse	Healthcare assistant	ENP/ACP	Junior Trainee (eg ST1-3)	Senior non-Consultant (eg ST4+)	Consultant	Other
CRP	☐	☐	☐	☐	☐	☐	☐
Procalcitonin	☐	☐	☐	☐	☐	☐	☐
RSV	☐	☐	☐	☐	☐	☐	☐
Influenza	☐	☐	☐	☐	☐	☐	☐
Group A Strep	☐	☐	☐	☐	☐	☐	☐
Blood gas	☐	☐	☐	☐	☐	☐	☐
Urinalysis	☐	☐	☐	☐	☐	☐	☐
Urinary BHCG	☐	☐	☐	☐	☐	☐	☐
Blood sugar	☐	☐	☐	☐	☐	☐	☐
Blood ketones	☐	☐	☐	☐	☐	☐	☐

For those where you answered other, please tell us who has responsibility for acting on these

____

Finally, some other general POCT questions...

How is POCT funded in your unit?

- ☐ All funded as part of ongoing service with sustainable longterm funding
- ☐ Some funded using temporary fund as part of a service evaluation
- ☐ Some funded through charitable funding and/or donations
- ☐ Some funded as part of an industry sponsored trial
- ☐ Other

Please tell us more about this POCT funding, if you know the details

____

Who is responsible for POCT governance in your unit(s)? Governance here refers to training, device maintenance, quality control, and managing any other issues that arise

	Laboratory team take full responsibility for governance	Clinical staff take some responsibility for governance, in conjunction with laboratory teams	Other	Not applicable
CRP	☐	☐	☐	☐
Procalcitonin	☐	☐	☐	☐
RSV	☐	☐	☐	☐
Influenza	☐	☐	☐	☐
Group A Strep	☐	☐	☐	☐
Blood Gas	☐	☐	☐	☐
Urinalysis	☐	☐	☐	☐
Urinary BHCG	☐	☐	☐	☐
Blood sugar	☐	☐	☐	☐
Ketones	☐	☐	☐	☐

Please tell us more about how POCT governance is provided

____

How and where are POC test results stored?

	Handwritten in clinical record	Manual entry in electronic record	Printed out and stuck in record	Auto upload to electronic system	Other	Not applicable
CRP	☐	☐	☐	☐	☐	☐
Procalcitonin	☐	☐	☐	☐	☐	☐
RSV	☐	☐	☐	☐	☐	☐
Influenza	☐	☐	☐	☐	☐	☐
Group A Strep	☐	☐	☐	☐	☐	☐
Blood gas	☐	☐	☐	☐	☐	☐
Urinalysis	☐	☐	☐	☐	☐	☐
Urinary BHCG	☐	☐	☐	☐	☐	☐
Blood sugar	☐	☐	☐	☐	☐	☐
Ketones	☐	☐	☐	☐	☐	☐

If you said other, please provide more detail here:

____

How easy would it be to to introduce a new POCT where you currently work?

Very difficult ——————————— Very easy

(Place a mark on the scale above)

What obstacles currently exist in your unit to the introduction of POCT?

- ☐ Difficulties with funding
- ☐ Nobody has time to run the test
- ☐ Nobody has time to perform the quality control testing
- ☐ Nobody will take responsibility for the governance of the test
- ☐ Evidence is lacking for POCT
- ☐ Other

Please expand on any/all of these obstacles...

____

If you've managed to introduce POC testing, please tell us what made that possible (ie what are the enablers to introducing such a test)?

____

Any other thoughts or comments on POCT are welcome - please scribble here...

____
