# Supplementary material for: Point-of-care testing in Paediatric settings in the UK and Ireland: a cross-sectional study
Source: BMC Emerg Med. 2022 Jan 11;22:6. doi: 10.1186/s12873-021-00556-7 (PMC8753865; doi:10.1186/s12873-021-00556-7)
Supplement: Supplementary file 2 — Additional file 2. [file 12873_2021_556_MOESM2_ESM.zip › Supplementary material- Tital page with author information.docx]

TITLE PAGE

Point-Of-Care Testing in Paediatric Settings in the UK and Ireland: a cross-sectional study

Authors

Meenu Pandey^1^, Mark D Lyttle^2,3^, Katrina Cathie^4^, Alasdair Munro^5^, Thomas Waterfield^6,7^, Damian Roland^1,8^

On behalf of GAPRUKI and PERUKI

1. Paediatric Emergency Medicine Leicester Academic (PEMLA) Group, Children’s Emergency Department, Leicester Royal Infirmary, Leicester, UK
2. Emergency Department, Bristol Royal Hospital for Children, Bristol, UK
3. Faculty of Health and Applied Sciences, University of the West of England, Bristol, UK
4. Department of Child Health, University Hospital Southampton NHS Foundation Trust
5. National Institute of Health Research Southampton Clinical Research Facility and Biomedical Research Centre, University Hospital Southampton NHS Foundation Trust
6. Wellcome-Wolfson Institute for Experimental Medicine, Queen's University Belfast
7. Emergency Department, Children’s Health Ireland, Temple Street, Dublin
8. SAPPHIRE Group, Health Sciences, Leicester University, Leicester, UK

Corresponding author: Dr. Damian Roland, Children’s Emergency Department, Leicester Royal Infirmary, Leicester, LE1 5WW. [dr98@leicester.ac.uk](mailto:dr98@leicester.ac.uk), 07950891367
