# Supplementary material for: Point-of-care testing in Paediatric settings in the UK and Ireland: a cross-sectional study
Source: BMC Emerg Med. 2022 Jan 11;22:6. doi: 10.1186/s12873-021-00556-7 (PMC8753865; doi:10.1186/s12873-021-00556-7)
Supplement: Supplementary file 2 — Additional file 2. [file 12873_2021_556_MOESM2_ESM.zip › Supplementary material.docx]

Table 5. Staff members who perform each POCT in different acute paediatric settings

|  |  | Blood sugar | Urinalysis | Blood gas | Blood Ketones | Urinary hCG | Influenza | RSV | CRP | Group A Strep | Procalcitonin | Total |
| --- | --- | --- | --- | --- | --- | --- | --- | --- | --- | --- | --- | --- |
| Clinical Nurse, n | Total | 135/705 | 134/705 | 107/705 | 120/705 | 115/705 | 45/705 | 39/705 | 6/705 | 4/705 | 0/705 | 705/705 |
|  | ED | 72/395 | 70/395 | 64/395 | 66/395 | 66/395 | 29/395 | 23/395 | 3/395 | 2/395 | 0/395 | 395/395 |
|  | PAU | 27/131 | 27/131 | 17/131 | 23/131 | 20/131 | 7/131 | 7/131 | 2/131 | 1/131 | 0/131 | 131/131 |
|  | UCC | 5/23 | 5/23 | 3/23 | 3/23 | 5/23 | 1/23 | 1/23 | 0/23 | 0/23 | 0/23 | 23/23 |
|  | IP | 31/156 | 32/156 | 23/156 | 28/156 | 24/156 | 8/156 | 8/156 | 1/156 | 1/156 | 0/156 | 156/156 |
| Healthcare assistant, n | Total | 79/400 | 87/400 | 46/400 | 64/400 | 86/400 | 19/400 | 14/400 | 2/400 | 3/400 | 0/400 | 400/400 |
|  | ED | 47/256 | 60/256 | 35/256 | 39/256 | 54/256 | 12/256 | 7/256 | 1/256 | 1/256 | 0/256 | 256/256 |
|  | PAU | 12/70 | 20/70 | 5/70 | 10/70 | 13/70 | 4/70 | 4/70 | 1/70 | 1/70 | 0/70 | 70/70 |
|  | UCC | 3/12 | 3/12 | 0/12 | 1/12 | 3/12 | 1/12 | 1/12 | 0/12 | 0/12 | 0/12 | 12/12 |
|  | IP | 17/80 | 22/80 | 6/80 | 14/80 | 16/80 | 2/80 | 2/80 | 0/80 | 1/80 | 0/80 | 80/80 |
| ENP/ACP, n | Total | 71/435 | 85/435 | 86/435 | 70/435 | 70/435 | 22/435 | 17/435 | 11/435 | 3/435 | 0/435 | 435/435 |
|  | ED | 46/270 | 55/270 | 56/270 | 42/270 | 48/270 | 10/270 | 6/270 | 6/270 | 1/270 | 0/270 | 270/270 |
|  | PAU | 15/85 | 16/85 | 15/85 | 13/85 | 11/85 | 6/85 | 5/85 | 3/85 | 1/85 | 0/85 | 85/85 |
|  | UCC | 2/10 | 2/10 | 1/10 | 1/10 | 2/10 | 1/10 | 1/10 | 0/10 | 0/10 | 0/10 | 10/10 |
|  | IP | 14/76 | 12/76 | 14/76 | 14/76 | 9/76 | 5/76 | 5/76 | 2/76 | 1/76 | 0/76 | 76/76 |
| Junior Doctor, n | Total | 61/385 | 74/385 | 121/385 | 52/385 | 43/385 | 10/385 | 7/385 | 13/385 | 2/385 | 2/385 | 385/385 |
|  | ED | 28/214 | 49/214 | 65/214 | 22/214 | 30/214 | 6/214 | 4/214 | 7/214 | 2/214 | 1/214 | 214/214 |
|  | PAU | 16/77 | 13/77 | 23/77 | 15/77 | 6/77 | 1/77 | 0/77 | 3/77 | 0/77 | 0/77 | 77/77 |
|  | UCC | 1/10 | 3/10 | 2/10 | 0/10 | 2/10 | 1/10 | 1/10 | 0/10 | 0/10 | 0/10 | 10/10 |
|  | IP | 16/84 | 9/84 | 31/84 | 15/84 | 5/84 | 2/84 | 2/84 | 3/84 | 0/84 | 1/84 | 84/84 |
| Consultant, n | Total | 54/332 | 59/332 | 109/332 | 47/332 | 34/332 | 9/332 | 5/332 | 11/332 | 2/332 | 2/332 | 332/332 |
|  | ED | 24/184 | 39/184 | 61/184 | 20/184 | 23/184 | 5/184 | 3/184 | 6/184 | 2/184 | 1/184 | 184/184 |
|  | PAU | 15/70 | 10/70 | 22/70 | 14/70 | 5/70 | 1/70 | 0/70 | 3/70 | 0/70 | 0/70 | 70/70 |
|  | UCC | 2/13 | 3/13 | 3/13 | 1/13 | 2/13 | 1/13 | 1/13 | 0/13 | 0/13 | 0/13 | 13/13 |
|  | IP | 13/65 | 7/65 | 23/65 | 12/65 | 4/65 | 2/65 | 1/65 | 2/65 | 0/65 | 1/65 | 65/65 |
| Other, n | Total | 2/16 | 5/16 | 2/16 | 3/16 | 2/16 | 0/16 | 2/16 | 0/16 | 0/16 | 0/16 | 16/16 |
|  | ED | 1/8 | 2/8 | 1/8 | 2/8 | 1/8 | 0/8 | 1/8 | 0/8 | 0/8 | 0/8 | 8/8 |
|  | PAU | 1/3 | 1/3 | 0/3 | 1/3 | 0/3 | 0/3 | 0/3 | 0/3 | 0/3 | 0/3 | 3/3 |
|  | UCC | 0/4 | 2/4 | 1/4 | 0/4 | 1/4 | 0/4 | 0/4 | 0/4 | 0/4 | 0/4 | 4/4 |
|  | IP | 0/1 | 0/1 | 0/1 | 0/1 | 0/1 | 0/1 | 1/1 | 0/1 | 0/1 | 0/1 | 1/1 |

hCG- human Chorionic Gonadotrophin, RSV- Respiratory Syncytial Virus, CRP- C-reactive protein, ED- Emergency Department, PAU- Paediatric Assessment Unit, UCC- Urgent Care Centre, IP -Inpatient

Table 6. Staff who are responsible for acting on POCT results in different acute paediatric settings

|  |  | Blood sugar | Urinalysis | Blood gas analysis | Blood Ketones | Urinary hCG | Influenza | RSV | CRP | Group A Streptococcus | Procalcitonin | Total |
| --- | --- | --- | --- | --- | --- | --- | --- | --- | --- | --- | --- | --- |
| Clinical Nurse, n | Total | 97/361 | 59/361 | 33/361 | 62/361 | 44/361 | 34/361 | 32/361 | 0/361 | 0/361 | 0/361 | 361/361 |
|  | ED | 51/177 | 25/177 | 14/177 | 28/177 | 22/177 | 19/177 | 18/177 | 0/177 | 0/177 | 0/177 | 177/177 |
|  | PAU | 19/77 | 14/77 | 6/77 | 15/77 | 10/77 | 7/77 | 6/77 | 0/77 | 0/77 | 0/77 | 77/77 |
|  | UCC | 3/13 | 3/13 | 1/13 | 1/13 | 3/13 | 1/13 | 1/13 | 0/13 | 0/13 | 0/13 | 13/13 |
|  | IP | 24/94 | 17/94 | 12/94 | 18/94 | 9/94 | 7/94 | 7/94 | 0/94 | 0/94 | 0/94 | 94/94 |
| Healthcare assistant, n | Total | 10/53 | 13/53 | 4/53 | 8/53 | 8/53 | 7/53 | 3/53 | 0/53 | 0/53 | 0/53 | 53/53 |
|  | ED | 5/27 | 7/27 | 2/27 | 4/27 | 3/27 | 5/27 | 1/27 | 0/27 | 0/27 | 0/27 | 27/27 |
|  | PAU | 2/12 | 3/12 | 1/12 | 2/12 | 2/12 | 1/12 | 1/12 | 0/12 | 0/12 | 0/12 | 12/12 |
|  | UCC | 1/5 | 1/5 | 0/5 | 0/5 | 1/5 | 1/5 | 1/5 | 0/5 | 0/5 | 0/5 | 5/5 |
|  | IP | 2/9 | 2/9 | 1/9 | 2/9 | 2/9 | 0/9 | 0/9 | 0/9 | 0/9 | 0/9 | 9/9 |
| ENP/ACP, n | Total | 93/507 | 91/507 | 80/507 | 86/507 | 78/507 | 35/507 | 29/507 | 10/507 | 4/507 | 1/507 | 507/507 |
|  | ED | 60/327 | 59/327 | 51/327 | 57/327 | 53/327 | 22/327 | 17/327 | 5/327 | 2/327 | 1/327 | 327/327 |
|  | PAU | 16/88 | 16/88 | 15/88 | 14/88 | 12/88 | 6/88 | 5/88 | 3/88 | 1/88 | 0/88 | 88/88 |
|  | UCC | 2/10 | 2/10 | 1/10 | 1/10 | 2/10 | 1/10 | 1/10 | 0/10 | 0/10 | 0/10 | 10/10 |
|  | IP | 15/82 | 14/82 | 13/82 | 14/82 | 11/82 | 6/82 | 6/82 | 2/82 | 1/82 | 0/82 | 82/82 |
| Junior Trainee (eg ST1-3) , n | Total | 132/702 | 128/702 | 120/702 | 118/702 | 109/702 | 40/702 | 36/702 | 12/702 | 5/702 | 2/702 | 702/702 |
|  | ED | 70/382 | 67/382 | 64/382 | 63/382 | 63/382 | 24/382 | 21/382 | 6/382 | 3/382 | 1/382 | 382/382 |
|  | PAU | 26/134 | 26/134 | 23/134 | 23/134 | 19/134 | 7/134 | 6/134 | 3/134 | 1/134 | 0/134 | 134/134 |
|  | UCC | 3/15 | 3/15 | 2/15 | 2/15 | 3/15 | 1/15 | 1/15 | 0/15 | 0/15 | 0/15 | 15/15 |
|  | IP | 33/171 | 32/171 | 31/171 | 30/171 | 24/171 | 8/171 | 8/171 | 3/171 | 1/171 | 1/171 | 171/171 |
| Senior non-Consultant (eg ST4+), n | Total | 135/736 | 132/736 | 127/736 | 124/736 | 113/736 | 45/736 | 40/736 | 13/736 | 5/736 | 2/736 | 736/736 |
|  | ED | 72/407 | 70/407 | 69/407 | 66/407 | 66/407 | 29/407 | 24/407 | 7/407 | 3/407 | 1/407 | 407/407 |
|  | PAU | 27/141 | 27/141 | 25/141 | 25/141 | 20/141 | 7/141 | 6/141 | 3/141 | 1/141 | 0/141 | 141/141 |
|  | UCC | 4/18 | 4/18 | 2/18 | 2/18 | 4/18 | 1/18 | 1/18 | 0/18 | 0/18 | 0/18 | 18/18 |
|  | IP | 32/170 | 31/170 | 31/170 | 31/170 | 23/170 | 8/170 | 9/170 | 3/170 | 1/170 | 1/170 | 170/170 |
| Consultant, n | Total | 132/727 | 130/727 | 127/727 | 123/727 | 111/727 | 44/727 | 40/727 | 13/727 | 5/727 | 2/727 | 727/727 |
|  | ED | 71/403 | 69/403 | 69/403 | 65/403 | 65/403 | 29/403 | 24/403 | 7/403 | 3/403 | 1/403 | 403/403 |
|  | PAU | 24/130 | 25/130 | 23/130 | 24/130 | 18/130 | 6/130 | 6/130 | 3/130 | 1/130 | 0/130 | 130/130 |
|  | UCC | 4/20 | 4/20 | 3/20 | 3/20 | 4/20 | 1/20 | 1/20 | 0/20 | 0/20 | 0/20 | 20/20 |
|  | IP | 33/174 | 32/174 | 32/174 | 31/174 | 24/174 | 8/174 | 9/174 | 3/174 | 1/174 | 1/174 | 174/174 |
| Other, n | Total | 2/11 | 2/11 | 2/11 | 1/11 | 2/11 | 1/11 | 1/11 | 0/11 | 0/11 | 0/11 | 11/11 |
|  | ED | 0/1 | 0/1 | 1/1 | 0/1 | 0/1 | 0/1 | 0/1 | 0/1 | 0/1 | 0/1 | 1/1 |
|  | PAU | 0/0 | 0/0 | 0/0 | 0/0 | 0/0 | 0/0 | 0/0 | 0/0 | 0/0 | 0/0 | 0/0 |
|  | UCC | 2/10 | 2/10 | 1/10 | 1/10 | 2/10 | 1/10 | 1/10 | 0/10 | 0/10 | 0/10 | 10/10 |
|  | IP | 0/0 | 0/0 | 0/0 | 0/0 | 0/0 | 0/0 | 0/0 | 0/0 | 0/0 | 0/0 | 0/0 |

POCT-Point of Care Test, hCG- human Chorionic Gonadotrophin, RSV- Respiratory Syncytial Virus, CRP- C-reactive protein, ENP- Emergency nurse practitioner, ANP- Advanced nurse practitioner, ST- Specialty trainee
